# DNA Barcodes for the Northern European Tachinid Flies (Diptera: Tachinidae)

**DOI:** 10.1371/journal.pone.0164933

**Published:** 2016-11-04

**Authors:** Jaakko L. O. Pohjoismäki, Jere Kahanpää, Marko Mutanen

**Affiliations:** 1 University of Eastern Finland, Department of Environmental and Biological Sciences, P.O.Box 111, 80101, Joensuu, Finland; 2 University of Helsinki, Finnish Museum of Natural History, Helsinki, Finland; 3 Department of Genetics and Physiology, PO. Box 3000, 90014 University of Oulu, Oulu, Finland; CNRS, FRANCE

## Abstract

This data release provides *COI* barcodes for 366 species of parasitic flies (Diptera: Tachinidae), enabling the DNA based identification of the majority of northern European species and a large proportion of Palearctic genera, regardless of the developmental stage. The data will provide a tool for taxonomists and ecologists studying this ecologically important but challenging parasitoid family. A comparison of minimum distances between the nearest neighbors revealed the mean divergence of 5.52% that is approximately the same as observed earlier with comparable sampling in Lepidoptera, but clearly less than in Coleoptera. Full barcode-sharing was observed between 13 species pairs or triplets, equaling to 7.36% of all species. Delimitation based on Barcode Index Number (BIN) system was compared with traditional classification of species and interesting cases of possible species oversplits and cryptic diversity are discussed. Overall, DNA barcodes are effective in separating tachinid species and provide novel insight into the taxonomy of several genera.

## Introduction

The Tachinidae are one of the most species rich families of Diptera, with almost 10,000 described species worldwide [1]. Of some 880 species reported from Europe, 328 have been recorded from Finland [2]. The latter number includes the following recent additions to the Finnish fauna: *Parasetigena silvestris* (Robineau-Desvoidy), *Admontia maculisquama* (Zetterstedt), *Lecanipa bicincta* (Meigen), *Trigonospila ludio* (Zetterstedt), *Winthemia speciosa* (Egger), *Carcelia puberula* Mesnil, *Lydella thompsoni* Herting, *Peribaea setinervis* (Thomson), *Synactia parvula* (Rondani) and *Billaea fortis* (Rondani). *Siphona variata* Andersen has proved to be a misidentification and the species has been removed from the Finnish checklist.

Where known, all tachinids are obligate parasitoids of other arthropods and as such have great ecological importance [3]. As tachinid community size and structure are influenced by a number of biological variables, their species diversity offers a useful proxy to assess habitat intactness and quality [4–6]. Moreover, tachinids are important natural enemies of many ecologically important pest species, such as nun moths, *Lymantria* spp. (Lepidoptera: Lymantriidae) [7,8], the European corn borer, *Ostrinia nubilalis* (Hübner) (Lepidoptera: Pyralidae) [9,10] and earwigs, *Forficula* sp. (Dermaptera) [11].

Because of their species richness, morphological diversity and varying characters, many tachinid species are challenging to determine even for experts. Whereas the European fauna is rather well known, difficulties in classification and the poor quality of the early taxonomic work makes especially the determination of the tropical tachinid species impassable without the study of the type specimens [12]. While excellent resources into the identification of the European species and Palearctic genera are existing [13,14], DNA barcodes based on the 658 bp mitochondrial *cytochrome oxidase I* gene (*COI*) sequence [15] could prove to be valuable in helping non-specialists in species identification as well as enabling the identification of early developmental stages. The latter is especially interesting, as it permits the assessment of parasitoid diversity and the study of local food webs by sampling hosts [16,17]. Besides using *COI* barcodes to uncover cryptic host differentiation in tachinids [18], a comprehensive *COI* barcode library can provide rough identification of taxa even if the actual species identity remains unsolved. Because of their multiple utilities and ease of use, DNA barcodes have become an integral part of modern ecology [19].

The presented dataset provides reference barcodes for 366 mainly north European tachinids. The barcode library has been collected as a part of the Finnish Barcode of Life (FinBOL, www.finbol.org) initiative and represents projects opening data release as well as the first comprehensive collection of DNA barcodes for European tachinids. We explore the performance of DNA barcodes and Barcode Index Numbers (BINs) in discriminating species and discuss several species groups showing barcode-sharing or extraordinary intraspecific variation.

## Materials and Methods

A total of 1,136 specimens belonging to 397 species of Tachinidae were included in the analysis. The majority of the samples were from the personal collection of JLOP, supplemented by specimens donated or loaned by other researchers and institutions (**Table 1 and S1 Table**). J.P. identified the majority of the specimens, using the available literature [13,14,20–22] and, in doubtful cases, with the help of specialists mentioned in the acknowledgments. 814 specimens have been collected from Finland, 163 from Germany, 57 from France, 22 from Greece and 16 from Italy and lower numbers from several other countries. Majority of the species occur in the northern Europe, with the exception of some rare Palearctic species, which were included in the study as being possible the only opportunity to DNA barcode these species. Notably, many tachinid species have a wide distribution range, enabling us to directly compare for example Mediterranean populations with the Finnish. Full specimen details, storing institutions and GenBank accession numbers are provided in the **S1 Table**. Taxonomic and collection information as well as voucher photographs are also available through individual specimen pages within the public dataset DS-TACFI (dx.doi.org/10.5883/DS-TACFI) in the BOLD (Barcode of Life Data Systems, www.boldsystems.org) barcode data repository [dataset numbers are to be released upon the acceptance of the manuscript]. Larger sets of specimens were photographed in the University of Oulu and a single leg was removed and sent in a 96-well plate for DNA extraction and sequencing to the Canadian Center for DNA Barcoding (CCDB).

**Table 1:** List of species and the number of specimens from which at least a partial *COI* sequence was recovered. Abbreviations for the countries of origin: B–Belgium, CH–Switzerland, DE–Germany, E–Spain, F–France, FIN–Finland, GB–Great Britain, GR–Greece, I–Italy, NL–The Netherlands, RUS–Russia, SE–Sweden. Singleton countries are written in full. Length of the longest COI sequence together with the number of ambiguous bases is indicated. The list follows the taxonomic order of Herting & Dely-Draskovitz [29].

**EXORISTINAE Robineau-Desvoidy, 1863**			
**Identification**	**Country**	**N**	***COI* max. length**
*Exorista* sg. *Adenia* female	FIN, CH	2	658[0nt]
*Exorista mimula* (Meigen, 1824)	FIN	1	658[0nt]
*Exorista rustica* (Fallén, 1810)	FIN, DE	8	658[0nt]
*Exorista fasciata* (Fallén, 1820)	FIN, Serbia	3	658[0nt]
*Exorista larvarum* (Linnaeus, 1758)	FIN, DE	3	658[0nt]
*Exorista glossatorum* (Rondani, 1859)	GR	1	658[0nt]
*Exorista grandis* (Zetterstedt, 1844)	FIN	2	658[0nt]
*Exorista sorbillans* (Wiedemann, 1830)	E	1	658[0nt]
*Exorista deligata* Pandellé, 1896	FIN, GR	3	658[0nt]
*Chetogena acuminata* Rondani, 1859	Croatia	1	658[0nt]
*Chetogena tschorsnigi* Ziegler, 1999	FIN, P	2	658[0nt]
*Diplostichus janitrix* (Hartig, 1838)	FIN	1	658[0nt]
*Phorocera assimilis* (Fallén, 1810)	DE	3	658[0nt]
*Phorocera grandis* (Rondani, 1859)	DE	1	658[0nt]
*Phorocera obscura* (Fallén, 1810)	FIN, DE	5	658[0nt]
*Parasetigena silvestris* (Robineau-Desvoidy, 1863)	DE	1	658[0nt]
*Phorinia aurifrons* Robineau-Desvoidy, 1830	FIN	2	658[0nt]
*Bessa selecta* (Meigen, 1824)	FIN	2	658[0nt]
*Belida angelicae* (Meigen, 1824)	FIN	5	658[0nt]
*Meigenia dorsalis* (Meigen, 1824)	FIN, DE	4	658[0nt]
*Meigenia majuscula* (Rondani, 1859)	DE	1	658[0nt]
*Meigenia mutabilis* (Fallén, 1810)	FIN, DE	3	658[0nt]
*Zaira cinerea* (Fallén, 1810)	FIN	4	658[0nt]
*Gastrolepta anthracina* (Meigen, 1826)	B, DE	2	658[0nt]
*Trigonospila ludio (Zetterstedt*, *1849)*	FIN	1	658[0nt]
*Medina collaris* (Fallén, 1820)	FIN, DE	3	658[0nt]
*Medina* nr. *collaris*	FIN	4	658[0nt]
*Medina luctuosa* (Meigen, 1824)	FIN	6	658[0nt]
*Medina separata* (Meigen, 1824)	FIN	4	658[0nt]
*Istocheta longicornis* (Fallén, 1810)	SE, F	2	658[0nt]
*Staurochaeta albocingulata* (Fallén, 1820)	FIN	3	658[0nt]
*Lecanipa bicincta* (Meigen, 1824)	FIN	1	636[2nt]
*Leiophora innoxia* (Meigen, 1824)	DE	2	658[0nt]
*Admontia blanda* (Fallén, 1820)	FIN	4	658[0nt]
*Admontia grandicornis* (Zetterstedt, 1849)	FIN	2	658[0nt]
*Admontia maculisquama* (Zetterstedt, 1859)	FIN	1	658[0nt]
*Admontia seria* (Meigen, 1824)	CH	1	658[0nt]
*Oswaldia eggeri* (Brauer & Bergenstamm, 1889)	FIN	1	658[0nt]
*Oswaldia muscaria* (Fallén, 1810)	FIN	6	658[0nt]
*Oswaldia reducta* (Villeneuve, 1930)	FIN, DE	2	658[0nt]
*Oswaldia spectabilis* (Meigen, 1824)	FIN	3	658[0nt]
*Paracraspedothrix montivaga* Villeneuve, 1919	DE	1	407[0nt]
*Blondelia nigripes* (Fallén, 1810)	FIN, DE	7	658[0nt]
*Compsilura concinnata* (Meigen, 1824)	I	1	658[0nt]
*Vibrissina turrita* (Meigen, 1824)	NL	1	658[0nt]
*Acemya acuticornis* (Meigen, 1824)	I	1	658[0nt]
*Acemya rufitibia* (von Roser, 1840)	FIN, DE	2	658[0nt]
*Paratryphera barbatula* (Rondani, 1859)	FIN	2	658[0nt]
*Paratryphera bisetosa* (Brauer & Bergenstamm, 1891)	FIN, CH	2	658[0nt]
*Chetina setigena* Rondani, 1856	F	1	658[0nt]
*Rhaphiochaeta breviseta* (Zetterstedt, 1838)	FIN	1	658[0nt]
*Smidtia amoena* (Meigen, 1824)	FIN	5	658[0nt]
*Smidtia conspersa* (Meigen, 1824)	FIN, F, I	3	658[0nt]
*Winthemia cruentata* (Rondani, 1859)	FIN	2	658[0nt]
*Winthemia erythrura* (Meigen, 1838)	FIN, DE	3	658[0nt]
*Winthemia quadripustulata* (Fabricius, 1794)	FIN	3	658[0nt]
*Nemorilla floralis* (Fallen, 1810)	DE	1	658[0nt]
*Nemorilla maculosa* (Meigen, 1824)	FIN	3	658[0nt]
*Aplomya confinis* (Fallén, 1820)	FIN	4	658[0nt]
*Phebellia glauca* (Meigen, 1824)	FIN	3	658[0nt]
*Phebellia glaucoides* Herting, 1961	FIN	1	658[0nt]
*Phebellia margaretae* Bergström, 2005	FIN	1	658[0nt]
*Phebellia nigripalpis* (Robineau-Desvoidy, 1847)	SE	1	658[0nt]
*Phebellia pauciseta* (Villeneuve, 1908)	FIN	2	658[0nt]
*Phebellia strigifrons* (Zetterstedt, 1838)	FIN	1	658[0nt]
*Phebellia stulta* (Zetterstedt, 1844)	FIN	1	658[0nt]
*Phebellia villica* (Zetterstedt, 1838)	FIN	1	658[0nt]
*Nilea hortulana* (Meigen, 1824)	FIN	3	658[0nt]
*Nilea innoxia* Robineau-Desvoidy, 1863	FIN	3	658[0nt]
*Nilea rufiscutellaris* (Zetterstedt, 1859)	FIN	3	658[0nt]
*Tlephusa cincinna* (Meigen, 1859)	FIN	7	658[0nt]
*Epicampocera succincta* (Meigen, 1824)	FIN, DE	3	658[0nt]
*Phryxe erythrostoma* (Hartig, 1838)	FIN	3	658[0nt]
*Phryxe magnicornis* (Zetterstedt, 1838)	FIN	2	658[0nt]
*Phryxe nemea* (Meigen, 1824)	DE	1	407[0nt]
*Phryxe vulgaris* (Fallén, 1810)	FIN	4	658[0nt]
*Periarchiclops scutellaris* (Fallén, 1820)	FIN, I	3	658[0nt]
*Bactromyia aurulenta* (Meigen, 1824)	FIN	3	658[0nt]
*Pseudoperichaeta nigrolineata* (Stephens, 1853)	FIN	1	658[0nt]
*Pseudoperichaeta palesoidea* (*Robineau-Desvoidy*, 1830)	I	2	658[0nt]
*Lydella grisescens* Robineau-Desvoidy, 1830	F, DE	2	658[0nt]
*Lydella ripae* (Brischke, 1885)	FIN	4	658[0nt]
*Lydella stabulans* (Meigen, 1824)	FIN	4	658[0nt]
*Lydella thompsoni* Herting, 1959	FIN, RUS	2	658[0nt]
*Cadurciella tritaeniata* (Rondani, 1859)	FIN	3	658[0nt]
*Drino galii* (Brauer & Bergenstamm, 1891)	FIN	1	658[0nt]
*Drino inconspicua* (Meigen, 1830)	FIN	1	658[0nt]
*Drino lota* (Meigen, 1824)	FIN	2	658[0nt]
*Drino vicina* (Zetterstedt, 1849)	FIN	1	658[0nt]
*Hubneria affinis* (Fallén, 1810)	FIN, DE	4	658[0nt]
*Carcelia atricosta* Herting, 1961	FIN	2	658[0nt]
*Carcelia bombylans* Robineau-Desvoidy, 1830	FIN	2	658[0nt]
*Carcelia gnava* (Meigen, 1824)	FIN	1	658[0nt]
*Carcelia laxifrons* Villeneuve, 1912	FIN	2	658[0nt]
*Carcelia lucorum* (Meigen, 1824)	FIN, DE	3	658[0nt]
*Carcelia puberula* Mesnil, 1941	FIN	3	658[0nt]
*Carcelia rasa* (Macquart, 1849)	FIN	1	415[0nt]
*Carcelia tibialis* (Robineau-Desvoidy, 1863)	FIN	3	658[0nt]
*Senometopia excisa* (Fallén, 1820)	FIN	1	658[0nt]
*Senometopia intermedia* (Herting, 1960)	DE	1	658[0nt]
*Senometopia pollinosa* Mesnil, 1941	FIN	1	658[0nt]
*Senometopia separata* (Rondani, 1859)	FIN	1	658[0nt]
*Thecocarcelia acutangulata* (Macquart, 1850)	NL	1	658[0nt]
*Erycia furibunda* (Zetterstedt, 1844)	FIN	4	658[0nt]
*Erycia fatua* (Meigen, 1824)	F	1	658[0nt]
*Erycia nr*. *fatua*	Bulgaria	1	658[0nt]
*Xylotachina diluta* (Meigen, 1824)	FIN	1	617[0nt]
*Alsomyia capillata* (Rondani, 1859)	F	1	658[0nt]
*Platymya fimbriata* (Meigen, 1824)	FIN	6	658[0nt]
*Eumea linearicornis* (Zetterstedt, 1844)	FIN, DE	4	658[0nt]
*Eumea mitis* (Meigen, 1824)	FIN	2	658[0nt]
*Myxexoristops abietis* Herting, 1964	FIN	1	658[0nt]
*Myxexoristops arctica* (Zetterstedt, 1838)	FIN	1	658[0nt]
*Myxexoristops blondeli* (Robineau-Desvoidy, 1830)	DE	1	658[0nt]
*Myxexoristops bonsdorffi* (Zetterstedt, 1859)	DE	1	658[0nt]
*Myxexoristops stolida* (Stein, 1924)	FIN	1	658[0nt]
*Zenillia libatrix* (Panzer, 1798)	FIN	1	658[0nt]
*Zenillia nr*. *dolosa (Meigen*, *1824)*	FIN	1	576[0nt]
*Clemelis pullata* (Meigen, 1824)	F, DE, I	3	658[0nt]
*Pales pavida* (Meigen, 1824)	FIN, F	4	658[0nt]
*Pales peregrina* Herting, 1975	F	1	658[0nt]
*Phryno vetula* (Meigen, 1824)	DE	2	658[0nt]
*Cyzenis albicans* (Fallén, 1810)	FIN	2	658[0nt]
*Cyzenis jucunda* (Meigen, 1838)	FIN	4	658[0nt]
*Botria frontosa* (Meigen, 1824)	SE	1	654[0nt]
*Botria subalpina* Villeneuve, 1910	SE	3	658[0nt]
*Ceromasia rubrifrons* (Macquart, 1834)	FIN	1	658[0nt]
*Allophorocera ferruginea* (Meigen, 1824)	FIN	7	658[0nt]
*Allophorocera lapponica* Wood, 1974	FIN	1	658[0nt]
*Ocytata pallipes* (Fallén, 1820)	DE	2	658[0nt]
*Erynnia ocypterata* (Fallén, 1810)	FIN	8	658[0nt]
*Elodia ambulatoria* (Meigen, 1824)	FIN	3	658[0nt]
*Sturmia bella* (Meigen, 1824)	F	2	658[0nt]
*Blepharipa pratensis* (Meigen, 1824)	DE	1	658[0nt]
*Masicera pavoniae (Robineau-Desvoidy*, *1830)*	B	1	658[0nt]
*Masicera silvatica (Fallen*, *1810)*	F	1	658[0nt]
*Prosopea nigricans* (Egger, 1861)	FIN	1	658[0nt]
*Hebia flavipes* Robineau-Desvoidy, 1830	FIN	2	658[0nt]
*Frontina laeta* (Meigen, 1824)	FIN	4	658[0nt]
*Thelymorpha marmorata* (Fabricius, 1805)	CH	1	658[0nt]
*Baumhaueria goniaeformis* (Meigen, 1824)	Hungary	1	658[0nt]
*Brachicheta strigata* (Meigen, 1824)	FIN	2	658[0nt]
*Gonia bimaculata* Wiedemann, 1819	Portugal	1	477[1nt]
*Gonia capitata* (De Geer, 1776)	F	1	658[0nt]
*Gonia divisa* Meigen, 1826	FIN	4	658[0nt]
*Gonia ornata* Meigen, 1826	FIN	5	658[0nt]
*Gonia picea* (Robineau-Desvoidy, 1830)	FIN, DE	6	658[0nt]
*Onychogonia cervini* (Bigot, 1881)	FIN	2	658[0nt]
*Onychogonia flaviceps* (Zetterstedt, 1838)	FIN	2	658[0nt]
*Spallanzania hebes* (Fallén, 1820)	DE	2	658[0nt]
**TACHININAE Robineau-Desvoidy, 1830**			
**Identification**	**Country**	**N**	***COI* max. length**
*Lydina aenea* (Meigen, 1824)	FIN	2	658[0nt]
*Lypha dubia* (Fallén, 1810)	FIN	5	658[0nt]
*Lypha ruficauda* (Zetterstedt, 1838)	FIN	3	658[0nt]
*Tachina canariensis* (Macquart, 1839)	E	1	658[0nt]
*Tachina fera* (Linnaeus, 1761)	FIN, F, DE	13	658[0nt]
*Tachina grossa* (Linnaeus, 1758)	FIN	2	658[0nt]
*Tachina lurida* (Fabricius, 1781)	F, DE	2	658[0nt]
*Tachina magnicornis* (Zetterstedt, 1844)	DE	1	658[0nt]
*Tachina* nr. *magnicornis*	FIN	10	658[0nt]
*Tachina nupta* (Rondani, 1859)	DE	1	658[0nt]
*Tachina ursina* (Meigen, 1824)	FIN	2	658[0nt]
*Nowickia alpina* (Zetterstedt, 1844)	FIN	3	658[0nt]
*Nowickia ferox* (Panzer, 1809)	DE, F	3	658[0nt]
*Nowickia marklini* (Zetterstedt, 1838)	FIN	5	658[0nt]
*Peleteria aenea* (Staeger, 1849)	Greenland	1	658[0nt]
*Peleteria prompta* (Meigen, 1824)	CH	1	658[0nt]
*Peleteria rubescens* Robineau-Desvoidy, 1830	FIN, F, I	3	658[0nt]
*Peleteria ruficornis* (Macquart, 1835)	FIN	1	658[0nt]
*Peleteria varia* (Fabricius, 1794)	F	3	630[0nt]
*Germaria angustata* (Zetterstedt, 1844)	GB	2	658[0nt]
*Linnaemya bergstroemi* Pohjoismäki & Haarto, 2015	FIN	1	658[0nt]
*Linnaemya haemorrhoidalis* (Fallén, 1810)	Armenia	1	658[0nt]
*Linnaemya picta* (Meigen, 1824)	DE	2	658[0nt]
*Linnaemya rossica* Zimin, 1954	FIN	2	658[0nt]
*Linnaemya tessellans* (Robineau-Desvoidy, 1830)	DE	2	658[0nt]
*Linnaemya vulpina* (Fallén, 1810)	FIN	2	658[0nt]
*Chrysosomopsis aurata* (Fallén, 1820)	SE	1	658[0nt]
*Panzeria laevigata* (Meigen, 1838)	NL	1	658[0nt]
*Panzeria puparum* (Fabricius, 1794)	FIN	2	658[0nt]
*Panzeria rudis* (Fallén, 1810)	FIN	8	658[0nt]
*Panzeria vagans* (Meigen, 1824)	DE	2	658[0nt]
*Appendicia truncata* (Zetterstedt, 1838)	FIN	4	658[0nt]
*Eurithia anthophila* (Robineau-Desvoidy, 1830)	FIN	6	658[0nt]
*Eurithia caesia* (Fallén, 1810)	F	2	658[0nt]
*Eurithia connivens* (Zetterstedt, 1844)	FIN	5	658[0nt]
*Eurithia consobrina* (Meigen, 1824)	FIN	2	658[0nt]
*Eurithia vivida* (Zetterstedt, 1838)	FIN, I, CH	4	658[0nt]
*Hyalurgus crucigerus* (Zetterstedt, 1838)	FIN	3	658[0nt]
*Hyalurgus lucidus* (Meigen, 1824)	FIN	2	658[0nt]
*Nemoraea pellucida* (Meigen, 1824)	DE	1	658[0nt]
*Gymnocheta magna* (Fallén, 1810)	FIN	1	658[0nt]
*Gymnocheta viridis* (Fallén, 1810)	DE, FIN	5	658[0nt]
*Gymnocheta* nr. *viridis*	FIN	4	658[0nt]
*Zophomyia temula* (Scopoli, 1763)	FIN	1	541[0nt]
*Cleonice callida* (Meigen, 1824)	FIN	3	658[0nt]
*Cleonice keteli* Ziegler, 2000	RUS	1	658[0nt]
*Loewia adjuncta* Herting, 1971	GR	1	658[0nt]
*Loewia brevifrons* (Rondani, 1856)	F	1	658[0nt]
*Loewia erecta* Bergström, 2007	FIN	5	658[0nt]
*Loewia foeda* (Meigen, 1824)	FIN	2	658[0nt]
*Synactia parvula* (Rondani, 1861)	FIN, DE	3	658[0nt]
*Eloceria delecta* (Meigen, 1824)	FIN	1	658[0nt]
*Pseudopachystylum gonioides* (Zetterstedt, 1838)	FIN	4	658[0nt]
*Pelatachina tibialis* (Fallén, 1810)	FIN	4	658[0nt]
*Macquartia dispar* (Fallén, 1820)	FIN	2	658[0nt]
*Macquartia nudigena* Mesnil, 1972	FIN	4	658[0nt]
*Macquartia tenebricosa* (Meigen, 1824)	DE	1	658[0nt]
*Macquartia tessellum* (Meigen, 1824)	GR	1	658[0nt]
*Macquartia viridana* Robineau-Desvoidy, 1863	FIN	3	658[0nt]
*Anthomyiopsis nigrisquamata* (Zetterstedt, 1838)	FIN	2	658[0nt]
*Triarthria setipennis* (Fallén, 1810)	FIN, F, DE	3	658[0nt]
*Trichactia pictiventris* (Zetterstedt, 1855)	GR	1	658[0nt]
*Germariocheta clavata* Villeneuve, 1937	RUS	1	658[0nt]
*Neaera atra* Robineau-Desvoidy, 1850	GR	1	658[0nt]
*Phytomyptera cingulata* (Robineau-Desvoidy, 1830)	FIN, DE	3	658[0nt]
*Phytomyptera minutissima* (Zetterstedt, 1844)	FIN	4	658[0nt]
*Phytomyptera nigrina* (Meigen, 1824)	FIN	1	658[0nt]
*Phytomyptera nigroaenea* (Herting, 1968)	FIN	1	658[0nt]
*Phytomyptera zonella* (Zetterstedt, 1844)	FIN	3	658[0nt]
*Graphogaster dispar* (Brauer & Bergenstamm, 1889)	FIN	1	658[0nt]
*Graphogaster nigrescens* Herting, 1971	FIN	1	658[0nt]
*Graphogaster vestita* Rondani, 1868	F	1	658[0nt]
*Ceromya bicolor* (Meigen, 1824)	FIN	2	658[0nt]
*Ceromya silacea* (Meigen, 1824)	FIN	5	658[0nt]
*Actia crassicornis* (Meigen, 1824)	FIN	1	658[0nt]
*Actia infantula* (Zetterstedt, 1844)	NL, GB	4	658[0nt]
*Actia lamia* (Meigen, 1838)	FIN, DE	6	658[0nt]
*Actia maksymovi* Mesnil, 1952	FIN	1	658[0nt]
*Actia nigroscutellata* Lundbeck, 1927	FIN	2	658[0nt]
*Actia pilipennis* (Fallén, 1810)	FIN, F, DE	4	658[0nt]
*Peribaea hertingi* Andersen, 1996	FIN	2	658[0nt]
*Peribaea setinervis* (Thomson, 1869)	NL	1	658[0nt]
*Peribaea tibialis* (Robineau-Desvoidy, 1851)	DE	1	655[3nt]
*Ceranthia abdominalis* Robineau-Desvoidy, 1830	FIN	2	658[0nt]
*Ceranthia lichtwardtiana* (Villeneuve, 1931)	FIN	2	658[0nt]
*Ceranthia tenuipalpis* (Villeneuve, 1921)	FIN	1	658[0nt]
*Aphantorhaphopsis verralli* (Wainwright, 1928)	FIN	1	658[0nt]
*Siphona boreata* Mesnil, 1960	FIN	2	658[0nt]
*Siphona collini* Mesnil, 1960	FIN	5	658[0nt]
*Siphona confusa* Mesnil, 1961	FIN	4	658[0nt]
*Siphona cristata* (Fabricius, 1805)	FIN	1	658[0nt]
*Siphona flavifrons* (Staeger, 1849)	FIN	7	635[0nt]
*Siphona geniculata* (De Geer, 1776)	FIN, DE	15	658[0nt]
*Siphona grandistyla* Pandelle, 1894	Austria	1	658[0nt]
*Siphona hokkaidensis* Mesnil, 1957	SE	1	392[0nt]
*Siphona ingerae* Andersen, 1982	FIN	3	658[0nt]
*Siphona maculata* (Staeger, 1849)	FIN	2	658[0nt]
*Siphona nigricans* (Villeneuve, 1930)	FIN	1	658[0nt]
*Siphona paludosa* Mesnil, 1960	FIN	11	658[0nt]
*Siphona pauciseta* Rondani, 1865	FIN	7	658[0nt]
*Siphona rossica* Mesnil, 1961	FIN	1	658[0nt]
*Siphona setosa* Mesnil, 1960	FIN	5	658[0nt]
*Siphona subarctica* Andersen, 1996	FIN, Norway	4	658[0nt]
*Aphria longilingua* Rondani, 1861	CH	1	658[0nt]
*Aphria longirostris* (Meigen, 1824)	FIN	1	658[0nt]
*Bithia acanthophora* (Rondani, 1861)	F	1	658[0nt]
*Bithia spreta* (Meigen, 1824)	DE	1	658[0nt]
*Demoticus plebejus* (Fallén, 1810)	FIN	2	658[0nt]
*Leskia aurea* (Fallén, 1820)	FIN	3	658[0nt]
*Solieria inanis* (Fallén, 1810)	FIN, CH	3	658[0nt]
*Solieria pacifica* (Meigen, 1824)	FIN	4	658[0nt]
*Mintho rufiventris* (Fallén, 1816)	F, DE	2	658[0nt]
*Minthodes picta* (Zetterstedt, 1844)	FIN	1	658[0nt]
*Microphthalma europaea* Egger, 1860	F	1	658[0nt]
*Dexiosoma caninum* (Fabricius, 1794)	FIN	3	658[0nt]
*Therobia leonidei* (Mesnil, 1965)	GR	1	633[0nt]
**DEXIINAE Macquart, 1834**			
**Identification**	**Country**	**N**	***COI* max. length**
*Trixa caerulescens* Meigen, 1824	FIN	8	658[0nt]
*Trixa conspersa* (Harris, 1776)	FIN	7	658[0nt]
*Billaea biserialis* (Portshinsky, 1881)	GR	2	658[0nt]
*Billaea fortis* (Rondani, 1862)	FIN	1	658[0nt]
*Billaea irrorata* (Meigen, 1826)	FIN	2	658[0nt]
*Billaea kolomyetzi* Mesnil, 1970	FIN	1	658[0nt]
*Billaea pectinata* (Meigen, 1826)	F	1	153[0nt]
*Billaea triangulifera* (Zetterstedt, 1844)	FIN	3	658[0nt]
*Dinera carinifrons* (Fallén, 1817)	I	1	658[0nt]
*Dinera ferina* (Fallén, 1816)	FIN, DE	4	658[0nt]
*Dinera grisescens* (Fallén, 1816)	FIN	2	471[0nt]
*Dinera* nr. *fuscata*	DE	5	658[0nt]
*Estheria petiolata* (Bonsdorff, 1866)	FIN, F	3	658[0nt]
*Estheria* nr. *petiolata*	GR	1	658[0nt]
*Dexia rustica* (Fabricius, 1775)	F	2	658[0nt]
*Prosena siberita* (Fabricius, 1775)	FIN	4	658[0nt]
*Zeuxia cinerea* Meigen, 1826	F	1	658[0nt]
*Zeuxia subapennina* Rondani, 1862	GR	1	658[0nt]
*Eriothrix argyreatus* (Meigen, 1824)	I, CH	2	658[0nt]
*Eriothrix prolixa* (Meigen, 1824)	F	1	658[0nt]
*Eriothrix rufomaculata* (De Geer, 1776)	FIN	7	658[0nt]
*Trafoia monticola* Brauer & Bergenstamm, 1893	FIN	2	658[0nt]
*Campylocheta fuscinervis* (Stein, 1924)	FIN	1	658[0nt]
*Campylocheta inepta* (Meigen, 1824)	FIN	2	658[0nt]
*Campylocheta praecox* (Meigen, 1824)	FIN	2	658[0nt]
*Blepharomyia angustifrons* Herting, 1971	FIN	4	658[0nt]
*Blepharomyia pagana* (Meigen, 1824)	FIN	2	658[0nt]
*Blepharomyia piliceps* (Zetterstedt, 1859)	FIN	3	658[0nt]
*Periscepsia carbonaria* (Panzer, 1798)	I, E	2	658[0nt]
*Periscepsia prunaria (Rondani*, *1861)*	FIN	2	658[0nt]
*Periscepsia nr*. *prunaria*	FIN	1	656[1nt]
*Periscepsia ringdahli (Villeneuve*, *1922)*	FIN	5	658[0nt]
*Periscepsia spathulata (Fallén*, *1820)*	FIN	3	658[0nt]
*Wagneria alpina* (Villeneuve, 1910)	FIN	1	658[0nt]
*Wagneria costata* (Fallén, 1820)	FIN	1	658[0nt]
*Kirbya moerens* (Meigen, 1830)	DE	2	658[0nt]
*Athrycia curvinervis* (Zetterstedt, 1844)	FIN	2	658[0nt]
*Athrycia impressa* (van der Wulp, 1869)	FIN	4	658[0nt]
*Athrycia trepida* (Meigen, 1824)	FIN	6	658[0nt]
*Voria ruralis* (Fallén, 1810)	FIN, DE	3	658[0nt]
*Voria nr*. *ruralis*	GR	1	658[0nt]
*Cyrtophleba ruricola* (Meigen, 1824)	FIN, DE	4	658[0nt]
*Cyrtophleba vernalis* (Kramer, 1917)	FIN	4	658[0nt]
*Klugia marginata* (Meigen, 1824)	FIN	2	658[0nt]
*Chaetovoria antennata* Villeneuve, 1920	FIN	1	611[0nt]
*Phyllomya volvulus* (Fabricius, 1794)	FIN	3	658[0nt]
*Thelaira nigrina* (Fallén, 1817)	FIN	5	658[0nt]
*Thelaira solivaga* (Harris, 1780)	F	2	658[0nt]
*Halidaya aurea* Egger, 1856	FIN	1	633[0nt]
*Stomina tachinoides* (Fallén, 1816)	F, E	2	658[0nt]
*Dufouria chalybeata* (Meigen, 1824)	FIN	3	658[0nt]
*Dufouria nigrita* (Fallén, 1810)	FIN	2	658[0nt]
*Rondania dimidiata* (Meigen, 1824)	FIN	1	658[0nt]
*Pandelleia albipennis* Villeneuve, 1934	GR	2	658[0nt]
*Microsoma exiguum* (Meigen, 1824)	FIN, F, DE	8	658[0nt]
*Freraea gagatea* Robineau-Desvoidy, 1830	FIN	2	658[0nt]
**PHASIINAE Robineau-Desvoidy, 1830**			
**Identification**	**Country**	**N**	***COI* max. length**
*Clytiomya continua* (Panzer, 1798)	F	1	623[0nt]
*Clytiomya* nr. *continua*	GR	1	658[0nt]
*Eliozeta pellucens* (Fallén, 1820)	F	1	658[0nt]
*Ectophasia crassipennis* (Fabricius, 1794)	DE	2	658[0nt]
*Subclytia rotundiventris* (Fallén, 1820)	FIN, DE	4	658[0nt]
*Gymnosoma clavatum* (Rohdendorf, 1947)	DE	2	658[0nt]
*Gymnosoma* nr. *costatum*	F	1	356[4nt]
*Gymnosoma dolycoridis* Dupuis, 1961	DE	6	658[0nt]
*Gymnosoma nudifrons* Herting, 1966	FIN	10	658[0nt]
*Gymnosoma rotundatum* (Linnaeus, 1758)	DE	1	658[0nt]
*Cistogaster globosa* (Fabricius, 1775)	FIN, DE	5	658[0nt]
*Opesia descendens* Herting, 1973	DE	1	658[0nt]
*Phasia aurigera* (Egger, 1860)	DE	2	658[0nt]
*Phasia aurulans* (Meigen, 1824)	FIN, DE	5	658[0nt]
*Phasia barbifrons* (Girschner, 1887)	FIN, DE	3	658[0nt]
*Phasia hemiptera* (Fabricius, 1794)	FIN, DE	2	658[0nt]
*Phasia mesnili* (Draber-Monko, 1965)	GR	1	658[0nt]
*Phasia obesa* (Fabricius, 1798)	FIN, DE, GR	10	658[0nt]
*Phasia pusilla* Meigen, 1824	FIN, GR	4	658[0nt]
*Phasia subcoleoptrata* (Linnaeus, 1767)	FIN	2	658[0nt]
*Xysta holosericea* (Fabricius, 1805)	F	1	658[0nt]
*Catharosia pygmaea* (Fallén, 1815)	FIN	1	658[0nt]
*Strongygaster celer* (Meigen, 1838)	E	1	658[0nt]
*Eulabidogaster setifacies* (Rondani, 1861)	DE	1	658[0nt]
*Leucostoma meridianum* (Rondani, 1868)	DE	1	658[0nt]
*Leucostoma simplex* (Fallén, 1815)	DE	1	658[0nt]
*Leucostoma tetraptera* (Meigen, 1824)	F	1	658[0nt]
*Clairvillia biguttata* (Meigen, 1824)	DE	1	658[0nt]
*Labigastera forcipata* (Meigen, 1824)	DE	2	658[0nt]
*Labigastera nitidula* (Meigen, 1824)	GR	1	658[0nt]
*Cinochira atra* Zetterstedt, 1945	FIN, NL	3	658[0nt]
*Lophosia fasciata* Meigen, 1824	FIN, DE	3	658[0nt]
*Cylindromyia auriceps* (Meigen, 1838)	DE	2	658[0nt]
*Cylindromyia bicolor* (Olivier, 1812)	F, DE	2	658[0nt]
*Cylindromyia brassicaria* (Fabricius, 1775)	FIN, F, DE	4	658[0nt]
*Cylindromyia interrupta* (Meigen, 1824)	FIN, DE	3	658[0nt]
*Cylindromyia pusilla* (Meigen, 1824)	FIN	3	658[0nt]
*Cylindromyia rufifrons* (Loew, 1844)	F	1	658[0nt]
*Hemyda obscuripennis* (Meigen, 1824)	DE	1	658[0nt]
*Hemyda vittata* (Meigen, 1824)	DE	1	658[0nt]
*Besseria anthophila* (Loew, 1871)	FIN	1	658[0nt]
*Phania curvicauda* (Fallén, 1820)	F	1	658[0nt]
*Phania funesta* (Meigen, 1824)	FIN, DE	3	658[0nt]
*Phania thoracica* Meigen, 1824	FIN	3	658[0nt]

The CCDB’s sequencing protocol is described in detail in deWaard et al. [23]. The primer pair LepF1 and LepF2 is primarily used to amplify the barcode region in Tachinid flies, but, in cases of failure, other primer sets were also attempted. Full primer details, laboratory reports, trace files, sequences and GenBank accession numbers can be retrieved from the sequence page of each record in BOLD. Before the sequences were uploaded to BOLD, several validation steps were conducted in CCDB to detect possible cases of contaminations, pseudogenes (NUMTs) and chimeric sequences. Sanger sequencing trace electropherograms were reviewed for quality, excising sequences associated with a mean trace quality “phred” score below 30 and where more than 10% of the bases showed a quality score below 20 after trimming of the primer sequences. Sequences that met these quality criteria were reviewed to excise those that are likely pseudogenes (NUMTs) or chimeric in origin. Pseudogenes were detected by comparing each sequence to a Hidden Markov Model [24] of the COI protein [25]. Some rare specimens were individually processed and sequenced following standard protocols in the Department of Environmental and Biological Sciences, University of Eastern Finland. Records were placed in the TACFI project that was administrated via the Barcode of Life Database (BOLD) www-interface using the available on-line tools.

Sequences were aligned using the standard BOLD nucleotide sequence alignment tool, and for full NJ analyses subsequently slightly edited using Mega 6 [26]. Since there is no length variation in the barcode fragment among the analyzed Tachinidae, alignment was straightforward. We used Neighbor Joining (NJ) method to examine and visualize genetic patterns revealed by the data. A tree constructed with BOLD under Kimura-2-parameter (K2P) substitution model and BOLD alignment was built primarily to show BIN assignments for the specimens and species. To test the effects of substitution model for the full data, the NJ analyses were conducted separately under the K2P and uncorrected p-distance models using Mega 6. Bootstrap node support values were calculated based on 500 replicates. Four specimens with less than 300 bp fragment were not included in Mega analyses because of lack of overlapping data with other specimens. Additionally, NJ trees for some specific species groups showing interesting patterns in barcodes were built in BOLD under K2P model and BOLD alignment. Distance statistics, including mean and maximum intraspecific divergence, distance to the nearest neighbor were retrieved using the Barcode gap analysis tool available in BOLD. Barcode Index Number (BIN) operational taxonomic units are automatically created in BOLD for sequences that fulfill minimum requirements. In short, sequences are initially clustered by employing a fixed 2.2% threshold of uncorrected p-distance, and refined into the final BINs by Markov clustering. Further details for BINs are provided in Ratnasingham and Hebert, 2013 [27].

## Results and Discussion

**Fig 1:**
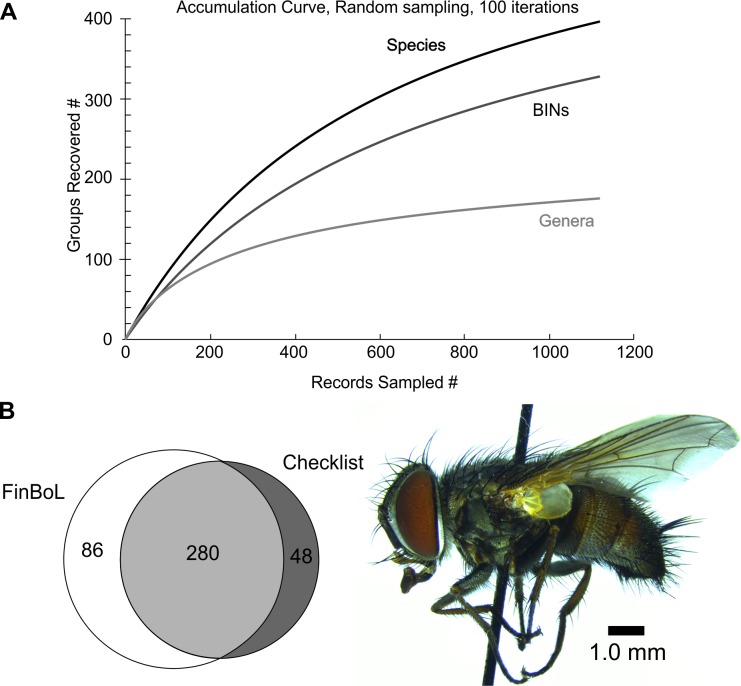
Overview of the tachinids sequenced in the FinBoL project. (A) Accumulation curve for the 366 species, corresponding 329 BINs. (B) The FinBoL project produced DNA barcodes for 280 Finnish tachinid species (85% of the species recorded in Finland) and for 86 non-Finnish species, which most are present in the adjacent countries. No samples or successful barcodes were obtained for 48 species on the Finnish checklist. Example species: Female *Carcelia bombylans* Robineau-Desvoidy, Espoo, Finland.

Over 200 bp DNA barcode was successfully obtained from 925 specimens representing of 366 species of Tachinids. Over 400 bp and over 600 bp sequence was recovered for 923 and 879 specimens, respectively. Sequences of sufficient length and quality for BIN assignment (usually >500 bp sequences of high quality) were assigned to 329 operational taxonomic units (OTUs) as based on Barcode Index Numbers (BINs) (**Fig 1 and S1 Fig, Table 1 and S1 Table**). Substitution model had some effects on the overall tree topology, especially orderings of higher clades, but little effect at the lower levels, i.e. sister-group relationships between and within the species (**S2 and S3 Figs**). 82% success rate for the specimens and 93% for the species can be regarded extraordinary, considering that >99% of the material were pinned dry specimens. The oldest pinned specimen from which full barcode sequence was retrieved, was collected in 1980. On one hand, we were unable to obtain PCR products from some species, such as *Rondania fasciata* (Macquart), despite numerous attempts with freshly collected material. We expect poor primer binding likely been involved in such cases. The data covers 85% of the Finnish fauna and all genera except *Policheta*, *Ligeria*, *Ligeriella* and *Peteina*. Additionally, we were able to cover several rare European genera, such as *Alsomyia*, *Trichactia*, *Pandelleia*, *Chaetovoria* and *Strongygaster*. The sequences for *Linnaemya*, *Lydina*, *Lypha*, *Peleteria*, *Nowickia* and *Tachina* have been released as a part of a previous study [28], but are listed also here for the completeness.

### Identification performance of the DNA barcodes

The included 366 species of Tachinidae show a mean minimum K2P divergence of 5.51% to the nearest neighbor (range 0.00–14.35%, SD = 0.62, SE = 0.03) (**S2 Table)**. This is on average 22.7 times the mean of maximum intraspecific variation (0.24%, range 0.00–7.58, SD = 3.51, SE = 0.18), demonstrating the general presence of a barcoding gap between species. This estimate of barcoding gap is however highly exaggerated by the presence of many singletons in our data. With singletons excluded, the mean barcoding gap drops to 13.8 times the mean of maximum intraspecific variation. This value is still an overestimate since the sampling was inadequate for providing a reliable estimate of total extent of intraspecific variation. Evidently, the true intraspecific variation is on average much less than the mean distance to the nearest neighbor. Moreover, the identification performance is negatively affected by several operational factors likely involved in our data as well [30]. For example, cases where a taxonomically accepted species actually consists of cryptic species highly exaggerates the estimate of intraspecific variation. Similarly, it is possible that our data include unjustified species, misidentifications, small sequencing and alignment errors, i.e. various operational factors, which all diminish the estimation of identification success of DNA barcodes.

The observed divergence of 5.51% between the species is slightly less than what has previously been reported in Lepidoptera (mean divergence among 2,577 species 5.73% [31]), and much less than in Coleoptera (mean divergence among 1,872 species 11.99% [32]) with similar sampling effort and geographic coverage. Comparisons of mean intraspecific divergences are slightly biased by different sampling, but differences between the insect groups remain evident, and are reflected to the protein level as well [31]. It has been suggested that *COI* evolution rate is generally correlated with the metabolic rate, with groups having high metabolic rate (such as Lepidoptera) tending to show slow evolution rate in *COI* compared to those having low metabolic rate (such as Coleoptera) [33]. This is in good accordance with our results, since many dipterans, including Tachinids, are strong fliers and are likely characterized with very high metabolic rate. Additionally, when compared to the ancient beetle families [34], Tachinidae are evolutionarily young, having underwent rapid diversification no earlier than the Oligocene [35] and therefore also explaining the large difference between the species divergence in the two groups.

Full barcode sharing (K2P distance to nearest neighbor = 0) was observed in 13 species pairs or triplets and between 28 species (7.36%) (**S2 Table**). In 53 species (14.4%), the divergence to the nearest neighbor was less than 1% and in 79 species (21.5%) less than 2%. These values are clearly higher than what was observed in beetles in the same region [32] since among 1,872 species of beetles only 1.6% showed full barcode sharing and 4.9% less than 2% divergence to the nearest neighbor. This difference is likely true and linked to the generally much larger interspecific distances in beetles than in tachinid flies.

### Taxa sharing BINs

**Fig 2:**
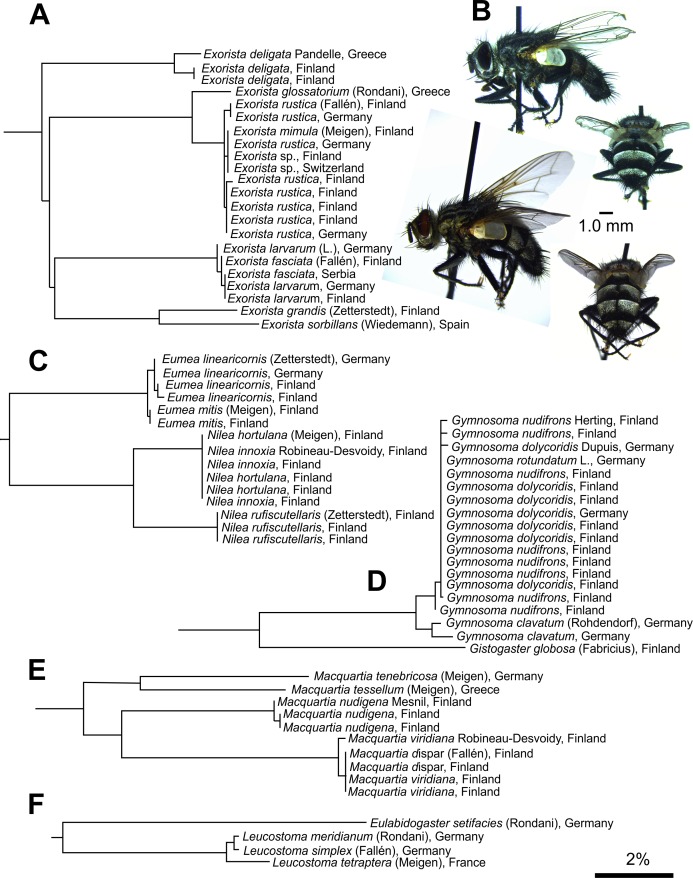
Examples of species or species complexes with poor BIN separation. (A) *Exorista mimula* (Meigen) *COI* sequence is embedded within the *E*. *rustica* (Fallén) sequences in the NJ trees. Same applies to *E*. *fasciata* (Fallén) and *E*. *larvarum* (L.), whereas similarly closely related *E*. *grandis* (Zetterstedt) and *E*. *sorbillans* (Wiedemann) are distinctly different. Notice also the differentiation between the Finnish and the Mediterranean specimens of *E*. *deligata* Pandelle. (B) Whereas *E*. *mimula* and *E*. *rustica* can be reliably determined only using male genital characters, *E*. *fasciata* (upper) and *E*. *larvarum* (lower) are clearly separable by various morphological characters and habitat preference. Other morphologically distinct species sharing BINs are (C) *Eumea linearicornis* (Zetterstedt) and *E*. *mitis* (Meigen), *Nilea hortulana* (Meigen) and *N*. *innoxia* Robineau-Desvoidy, (D) *Gymnosoma* spp. (E) *Macquartia dispar* (Fallén) and *M*. *viridiana* Robineau-Desvoidy as well as (F) *Leucostoma* spp. Scale bar: 2% sequence difference.

**Fig 3:**
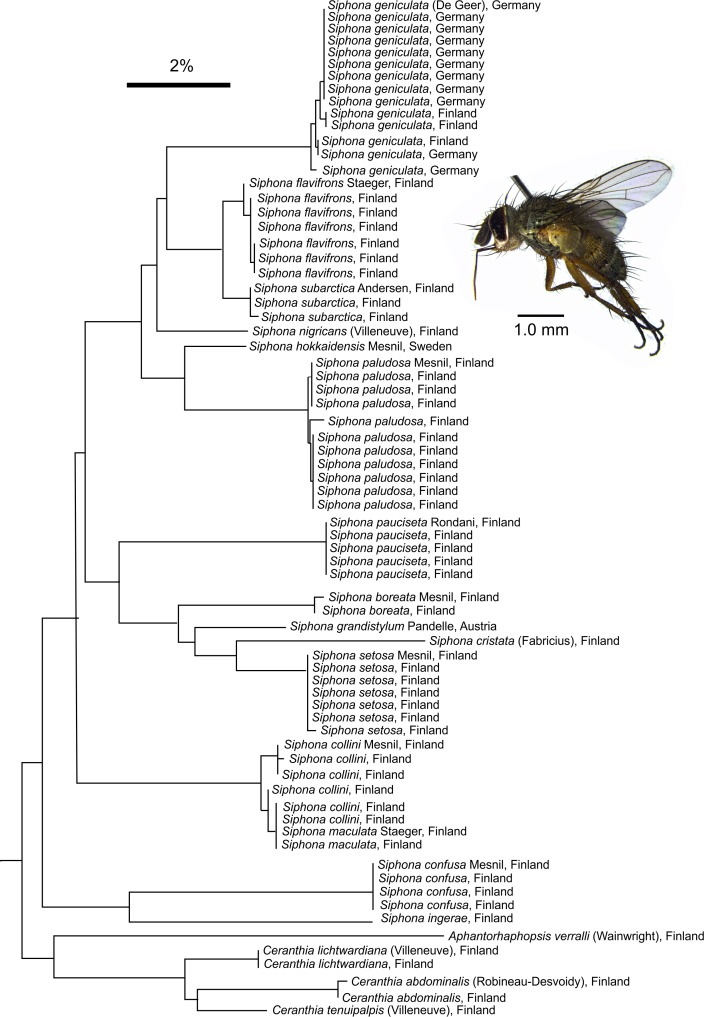
NJ tree of *Siphona* COI sequences. Contrary to the expectations, BINs have a good resolution in this morphologically variable genus, whose members are notoriously challenging to determine, only expectation is the *S*. *collini* Mesnil–*S*. *maculata* Staeger pair. Some duplicates omitted from the NJ tree for clarity, see **S2** and **S3 Figs** for the full data. Example species: Male *Siphona setosa* Mesnil, Jämijärvi, Finland.

Considering their recent evolutionary origin [35], BIN OTUs performed generally well for separating the studied taxa. However, some morphologically clearly separable species in *Exorista*, *Nilea*, *Eumea* (all Exoristinae), *Peleteria*, *Nowickia*, *Macquartia* (all Tachininae), *Gymnosoma* and *Leucostoma* (Phasiinae) share BIN as they had identical or nearly identical *COI* sequence (**Fig 2, S2 Table**). Interestingly, at least the members of *Exorista* sg. *Adenia* seem to be also poorly separable by nuclear genes [36]. Surprisingly, the species in morphologically difficult genus *Siphona*, had distinct BINs with the exception of *S*. *maculata* Staeger and *S*. *collini* Mesnil (**Fig 3**). BIN sharing occur in many taxa and may even be common in some groups [32,37], although the underlying mechanisms vary [38,39]. Whereas the *Exorista* species are undoubtedly evolutionary young, it is interesting that the Greenlandic *Peleteria aenea* Staeger and *P*. *rubescens* Robineau-Desvoidy from Mediterranean France are also almost inseparable (**S1–S3 Figs**, discussed previously in [28]). *Siphona collini* and *S*. *maculata* can be distinguished by a number of external characters, but for example their genital features are less descript and variable [40]. As they have a similar distribution with differing flight times, it is not impossible that the two could represent spring and high summer forms of the same species. It is clear that issues such as this can be only resolved by genome-wide analysis such as RAD sequencing [41] or meticulous study of the species’ biology.

### BIN variation: Geography and putative cryptic species

**Fig 4:**
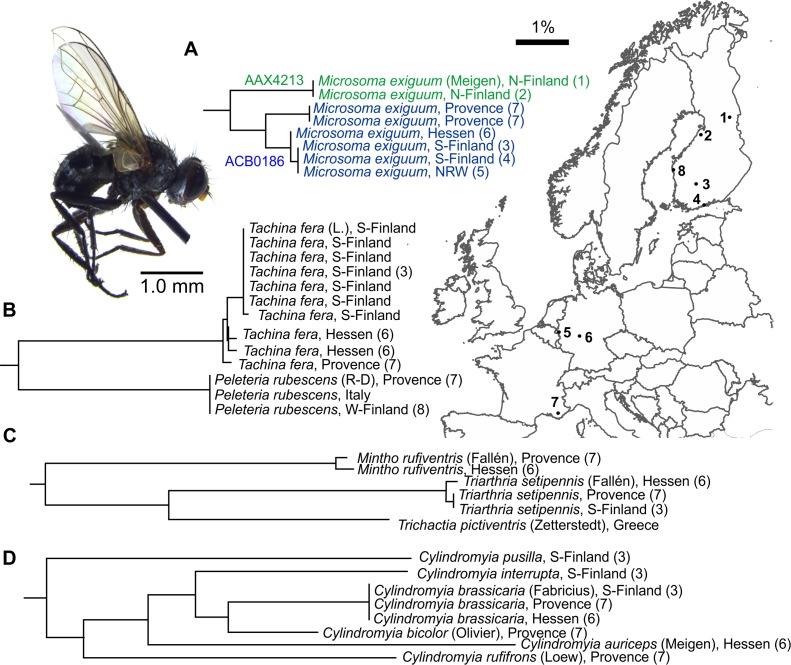
Geographic variation in tachinid *COI* sequences. (A) The northern Finnish (map locations 1–2) *Microsoma exiguum* (Meigen) belong to a different BIN cluster than the specimens from southern Finland, Central Europe (map locations 3–6) and Mediterranean France (map location 7). Note that the specimens from Provence represent a different haplogroup than the Central European ones, although the difference is not enough to split the BIN. Similar differentiation was not observed for (B) *Tachina fera* (L.), (C) *Mintho rufiventris* (Fallén), *Thriarthria setipennis* (Fallén) and (D) *Cylindromyia brassicaria* (Fabricius) collected from the same locations. Example species: Male *Microsoma exiguum*, Friedberg, Germany. Scale bar: 1% sequence difference.

While the aforementioned species exhibited barcode sharing, some species showed significant sequence divergence within or between different geographical regions. The most extreme example is *Microsoma exiguum* (Meigen) for which three distinct haplotypes with maximum divergence of 2.99% were detected; Mediterranean, Central- and Northern European (**Fig 4**). *M*. *exiguum* is the only known member of its genus in the Palearctic region. The flies are small (<3.0 mm) and live as parasitoids of adult weevils (Coleoptera: Curculionidae). Their small size alone could have implications on their dispersal ability, resulting in geographical differentiation. However, as the Central European haplotype (locality 5) was present also in southern Finland (localities 3 and 4), which is much more distant from Germany than the Mediterranean France, it is likely that the differentiation is explained by other biological factors, such as host specialization [42]. *Exorista deligata* Pandellé represents a similar case, although the difference and/or the sample size is not big enough to separate BINs (maximum intraspecific divergence 1.24%, n = 3) and that only two different haplotypes were found (**Fig 2**). *E*. *deligata* has an interesting discontinuous range, being present in the Mediterranean countries and Scandinavia, but absent from the Central Europe [43]. As far as known, they are specialized parasitoids of bagworm moths (Lepidoptera: Psychidae) and it is unlikely that the Finnish and Mediterranean subpopulations share any common host species [14]. As a comparison, *Peleteria rubescens* (Robineau-Desvoidy), *Tachina fera* (L.), *Thriathria setipennis* (Fallén) and *Cylindromyia brassicaria* (Fabricius) collected from the same locations exhibited less or no variation in their *COI* sequences (**Fig 4**). All these species are rather common across Europe and are likely to be generalists in their host use.

**Fig 5:**
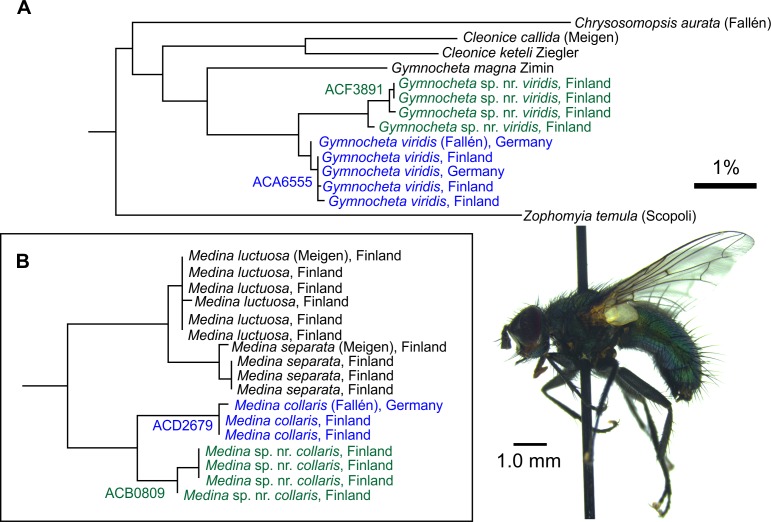
Intraspecific BIN splits in Finnish Tachinidae. (A) Finnish *Gymnocheta viridis* (Fallén) are split into two separate BIN clusters with a minimum divergence of 1.41% between them and a maximum divergence of 0.62% within the clusters. The *G*. sp. nr. *viridis* is also ecologically separable from the true *G*. *viridis* with all records being solely from the northern and eastern Finnish bog habitats, whereas the latter is almost purely a meadow species. (B) The Finnish *Medina collaris* (Fallén) specimens are similarly falling into two separate BINs. Coincidentally to *G*. *viridis* example also *M*. sp. nr. *collaris* are confined to bog habitats. Both *Medina* species have one rear bristle on their forelegs, a character state not present in other European representative of the genera. Example species: Male *Gymnosoma* sp. nr. *viridis*, Lieksa, Finland. Scale bar: 1% sequence difference.

*COI* barcodes also revealed deep intraspecific splits in the Finnish populations of *Gymnocheta viridis* (Fallén) and *Medina collaris* (Fallén), which seem to be also associated with habitat preference and morphology (JP, personal observations) (**Fig 5**). The identity of the different forms is currently under investigation. It should be noted that similar examples exist to lesser extent in some other genera, where variation within the species is not quite enough to differentiate BINs (**S1 Fig**).

### Possible taxonomic implications

**Fig 6:**
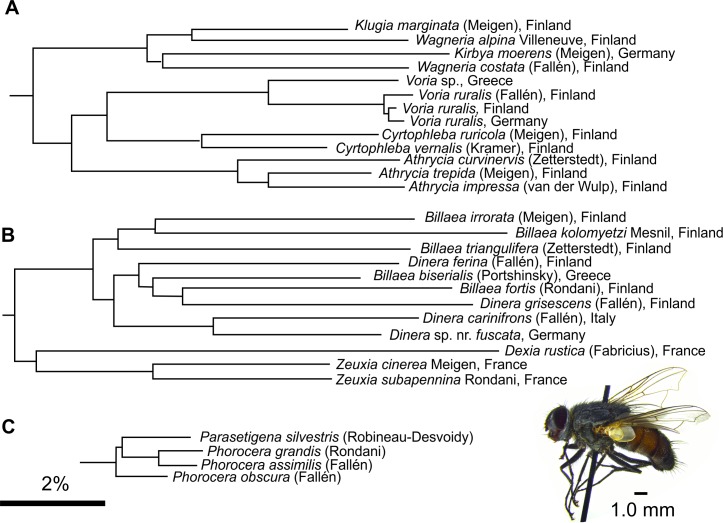
Possible taxonomic conflicts within tachinid genera. (A) *Kirbya moerens* (Meigen) is embedded within the *COI* sequences of the closely related *Wagneria*, whereas the other Voriini genera form their own clusters. *Voria* is thought only to be represented by *V*. *ruralis* (Fallén) in the Palearctic. However, the *COI* of a specimen from S-Agean Greece differs significantly (by 4.91%) from the northern European examples and could represent a species of its own. Similar to *Kirbya–Wagneria* case, also (B) *Billaea–Dinera* and (C) *Phorocera–Parasetigena* have mixed *COI* clusters. Example species: Male *Billaea kolomyetzi*, Ruokolahti, Finland. Scale bar: 2% sequence difference.

Although *COI* sequences are normally highly similar among the species within a genus, there are some occasions where the species from one genus are embedded among the species of another. As NJ can cluster unrelated sequences accidently, it is meaningful to compare only closely related taxa. Tachinids are notoriously rich in genera and can be that some of these sequence associations reflect unjustified splitting of genera by taxonomists. This is likely to be the case with *Wagneria-Kirbya* and *Billaea-Dinera* as well as *Phorocera* and *Parasetigena*, latter which originally belong to *Phorocera* (**Fig 6**). As of note, *Dinera* sp. nr. *fuscata* is a widespread species in Central Europe, which has been previously confused with *Dinera carinifrons* (Fallén). The European specimens differ from the Oriental *D*. *fuscata* Zhang & Shima and their taxonomic status needs to be solved [44]. The true *D*. *carinifrons* has apparently declined drastically and is probably extinct in Finland [2].

**Fig 7:**
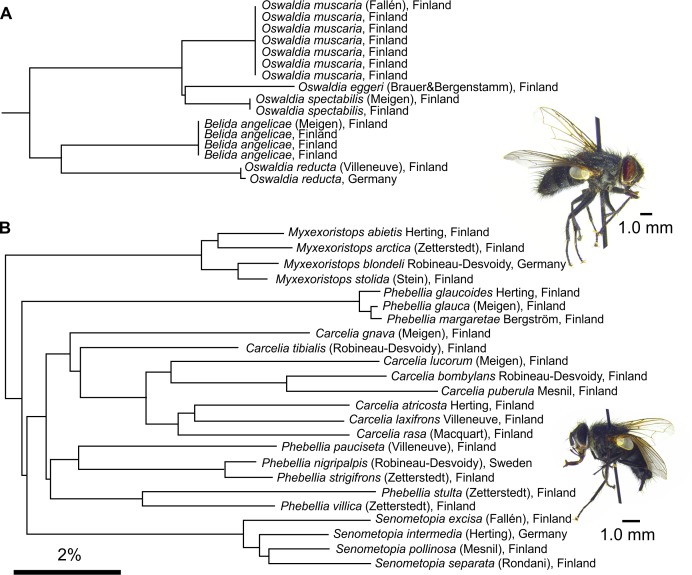
Deep divergence within tachinid genera. (A) *COI* from *Oswaldia reducta* (Villeneuve) has more sequence similarity with genera other than *Oswaldia*. (B) Species of *Phebellia* fall into two distantly related clusters, which also do not represent the proposed split of the genus into *Phebellia* s. str. and *Prooppia* [45].

Whereas the previous examples might indicate unjustified splitting of genera there are also opposite examples. For instance, *Oswaldia reducta* does not share much similarity with the other three species of *Oswaldia*, but is in all comparisons closer to other Blondelini, such as *Belida* (**Fig 7 and S1–S3** **Figs**). Similarly, *Phebellia* seems to be split into two distinct lineages. While *COI* can be useful in identifying possible taxonomical conflicts, the proper revision of the genera needs be based on additional genetic markers and morphological characters.

### Concluding Remarks

We provide here the first comprehensive collection of DNA barcodes for the European Tachinidae. Simultaneously the collection represents the first ever data release of Diptera from the FinBoL initiative. The DNA barcodes provided here permit the identification of the majority of the Finnish fauna and are likely to suffice for all of the common European species. Acknowledging the taxonomic difficulties, a great deal of care has been taken to confirm the species determinations and the data should provide a good reference for taxonomical and ecological studies using tachinids in the future. Importantly for tachinids, which are often unidentifiable as wet samples, pinned specimens proved to be perfectly adequate as source material for DNA barcoding.

## Supporting Information

S1 Fig BOLD taxon ID Tree, constructed with neighbor-joining method and under K2P evolutionary model, of all samples as taken from BOLD. BIN clusters given in different colours.
(PDF)

S2 Fig A neighbor-joining tree of near-full data constructed with Mega 6 under K2P model of nucleotide substitution. Node bootstrap support values based on 500 replicates are shown.
(EMF)

S3 Fig A neighbor-joining tree of near-full data constructed with Mega 6 under P-distance model of nucleotide substitution. Node bootstrap support values based on 500 replicates are shown.
(EMF)

S1 Table List of specimens in alphabetical order with sample location, BOLD process ID, BOLD sample ID and GenBank access number. The list includes also the failed specimens. Note that the taxonomy in BOLD follows O’Hara and Wood [45], treating *Ramonda* as the synonyme of *Periscepsia*.
(XLSX)

S2 Table Barcode cap analysis of tachinid species included in the study. Mean and maximum intraspecies variation, distance to the nearest neighbor (NN) and the nearest species are given.
(XLSX)
