# Supplementary figures and images for: DNA Barcodes for the Northern European Tachinid Flies (Diptera: Tachinidae)
Source: PLoS One. 2016 Nov 4;11(11):e0164933. doi: 10.1371/journal.pone.0164933 (PMC5096672; doi:10.1371/journal.pone.0164933)

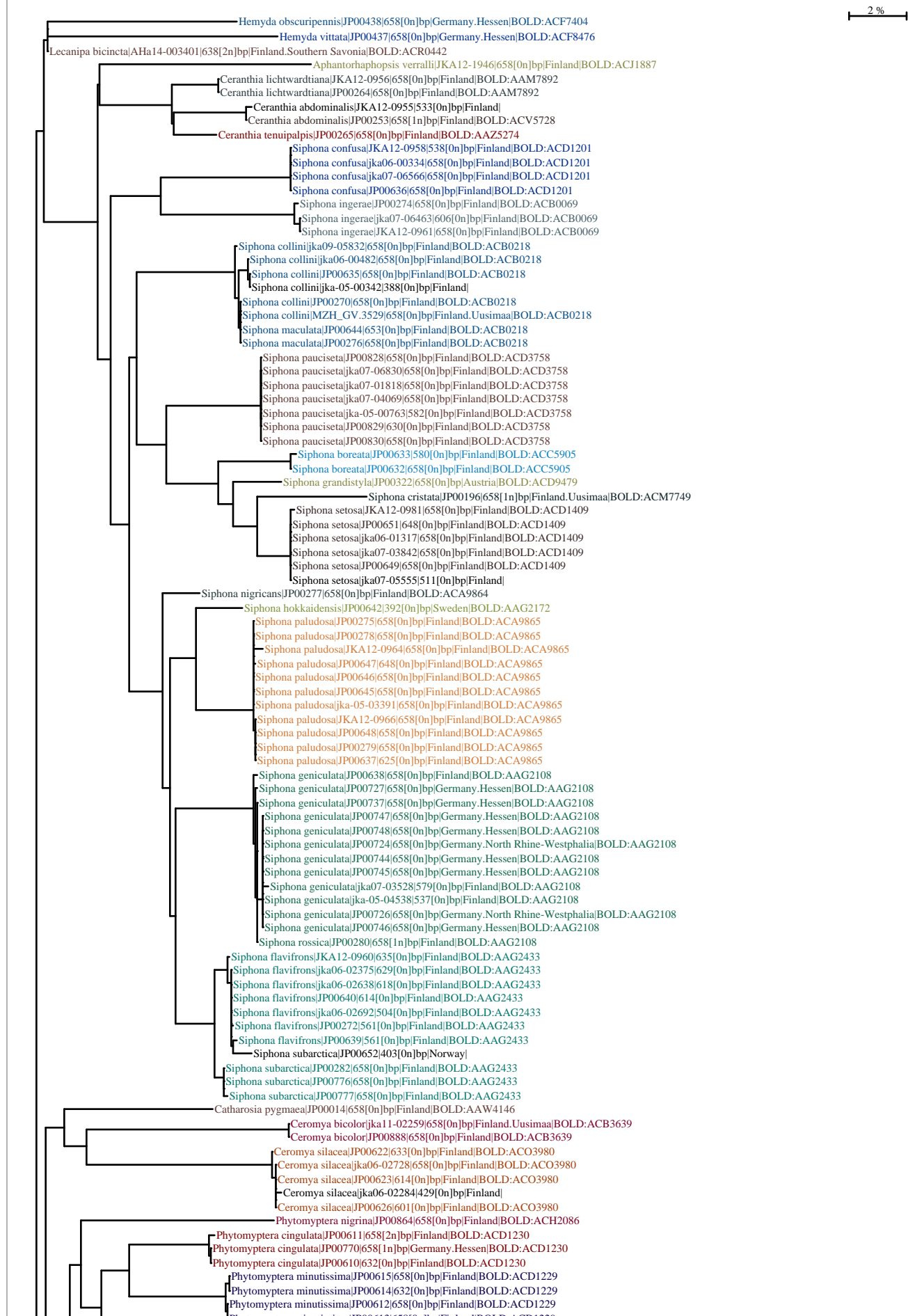

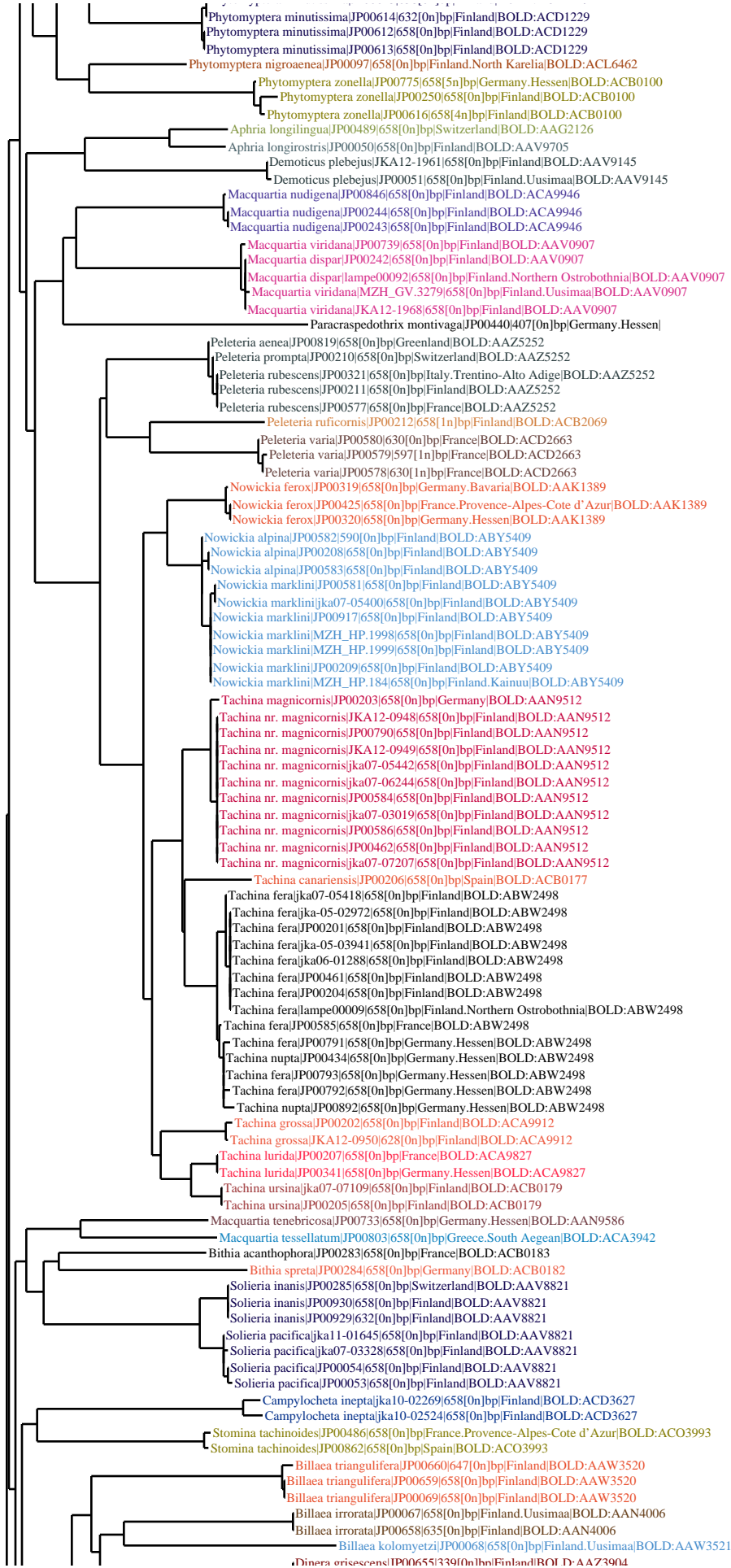

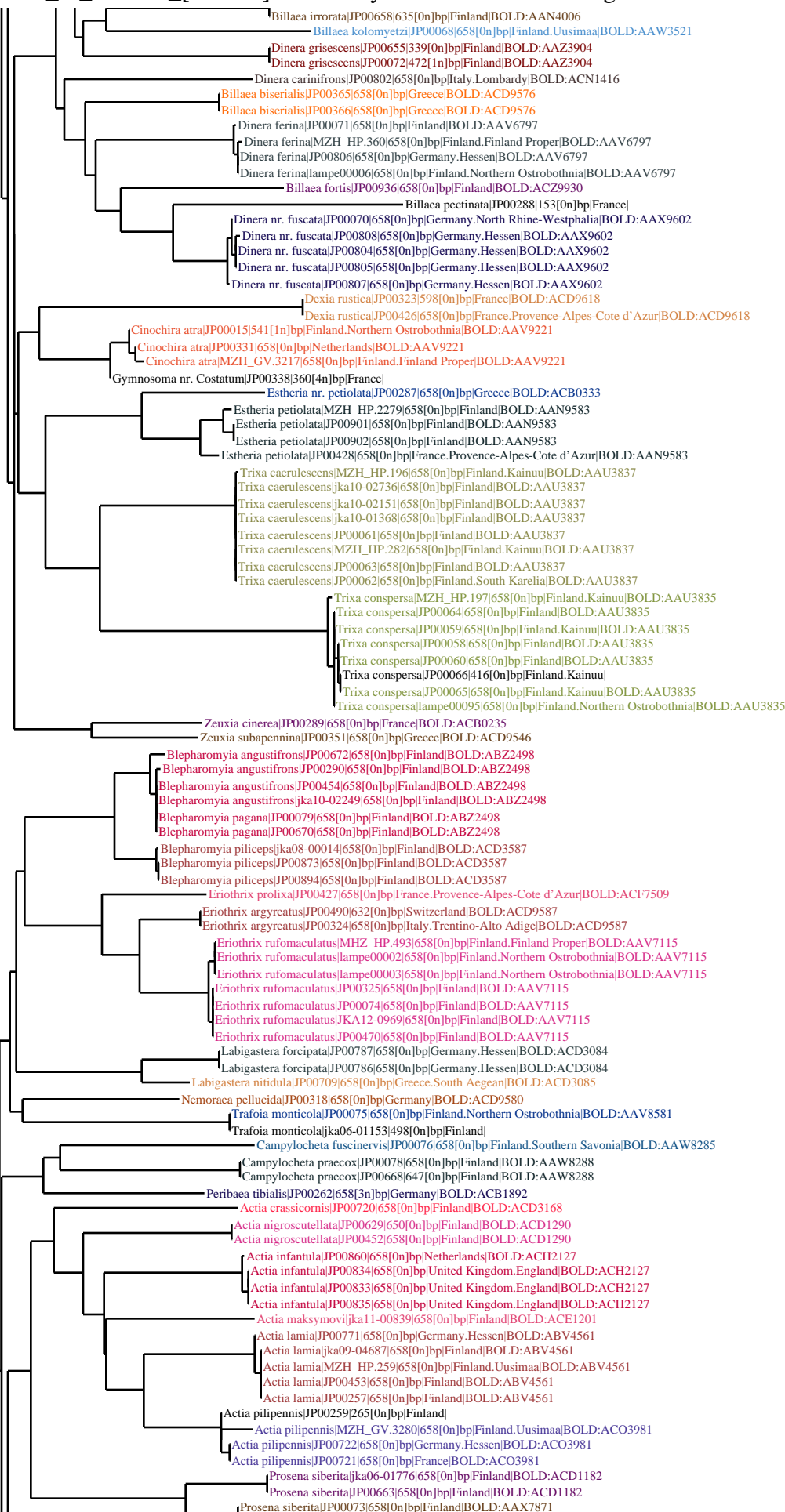

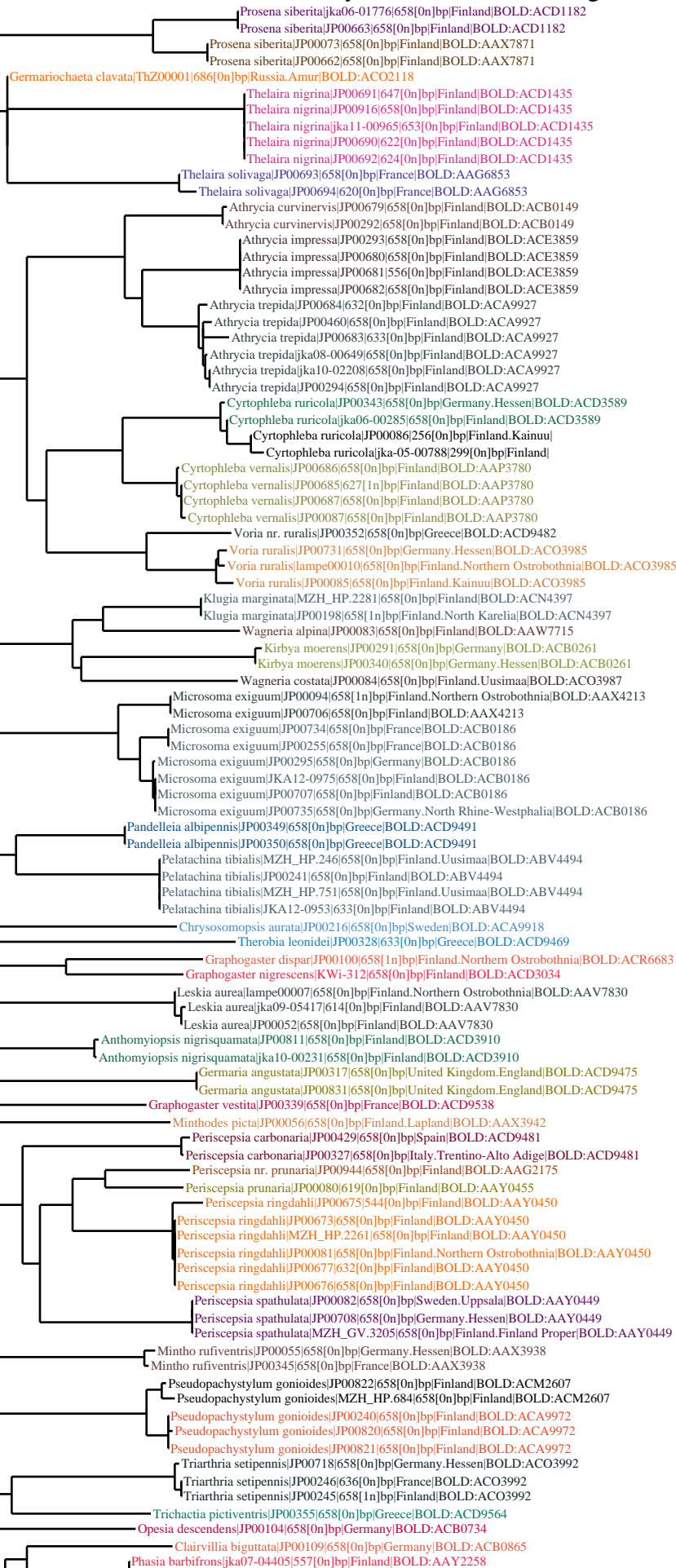

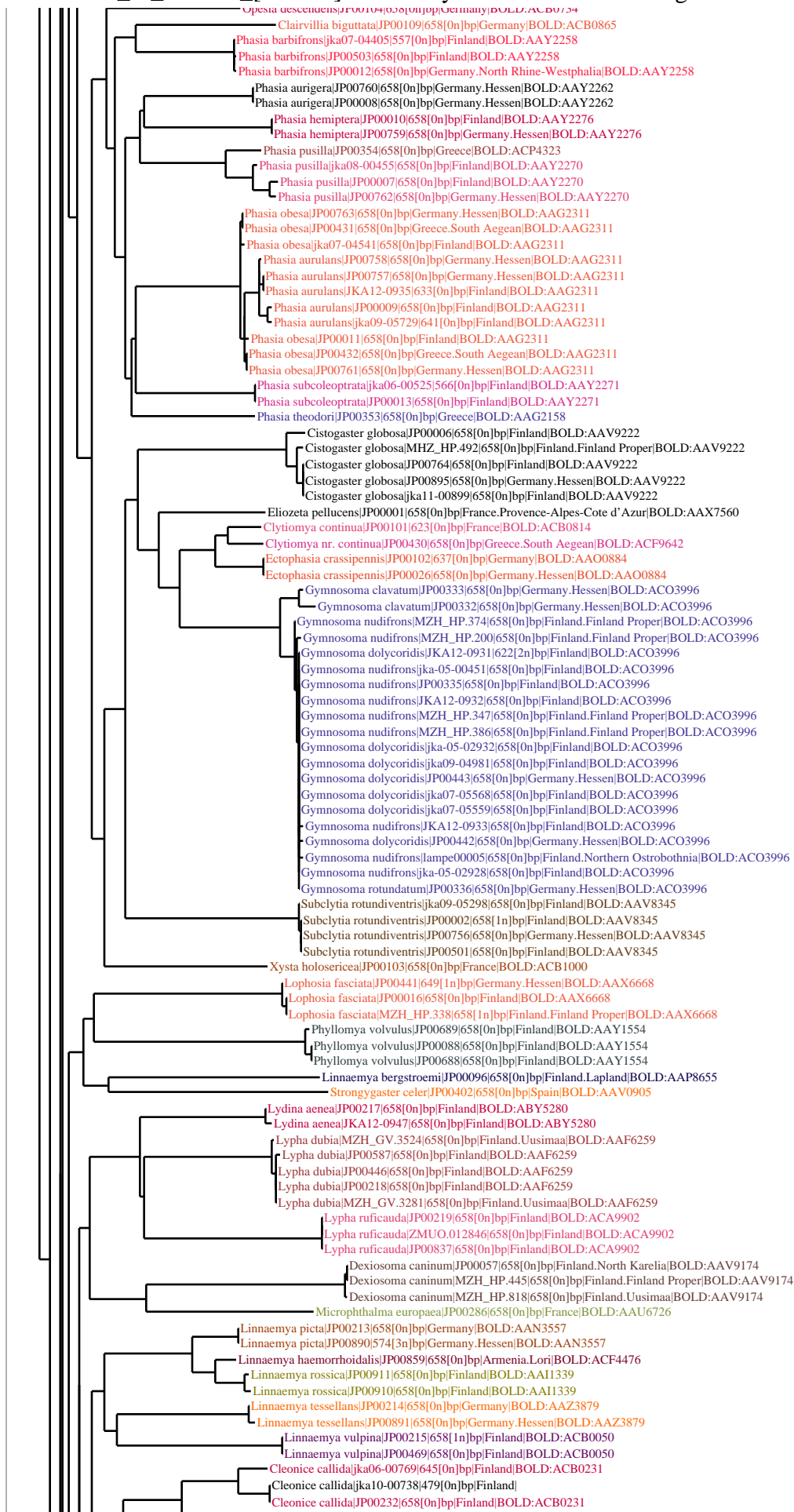

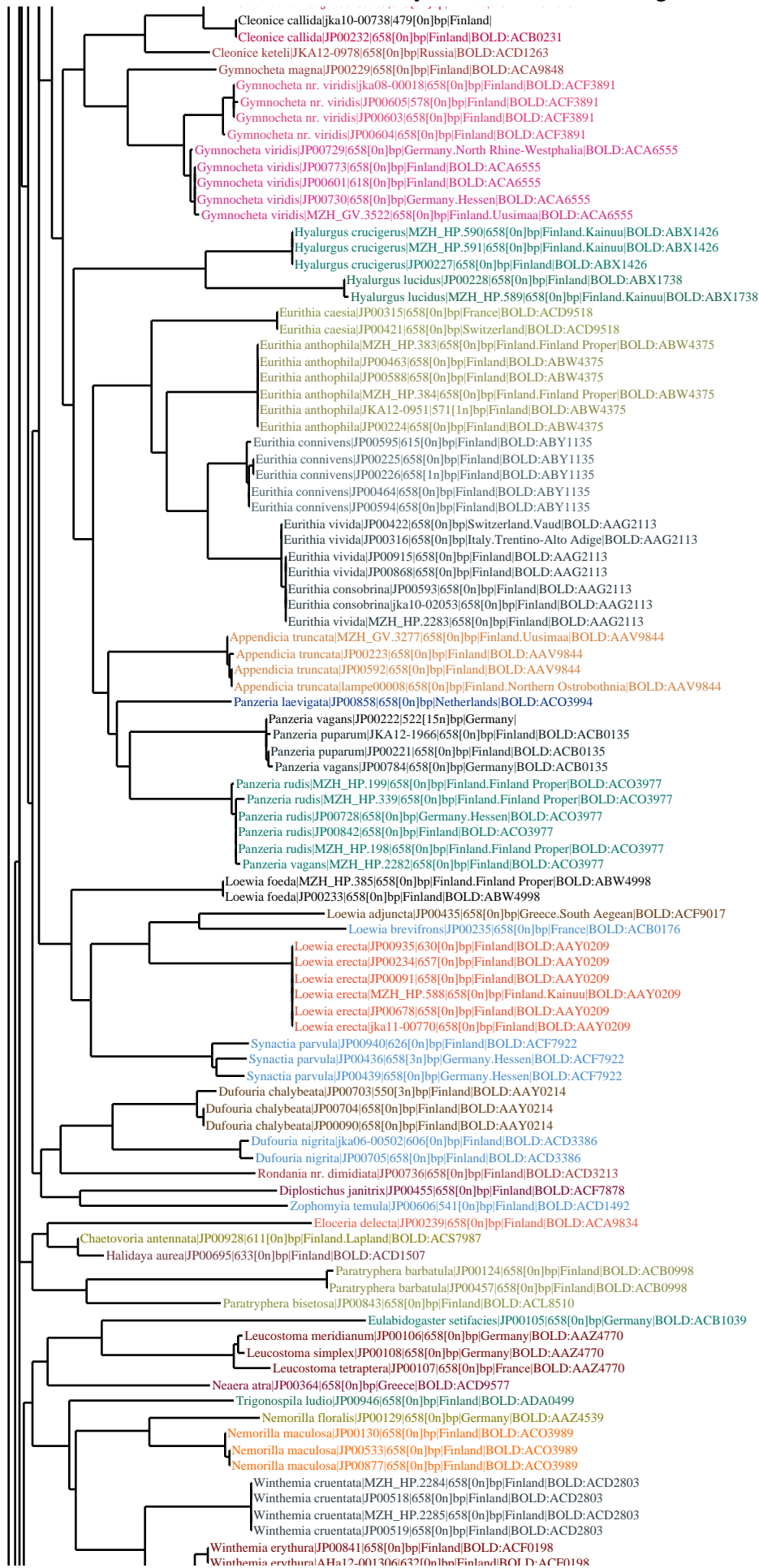

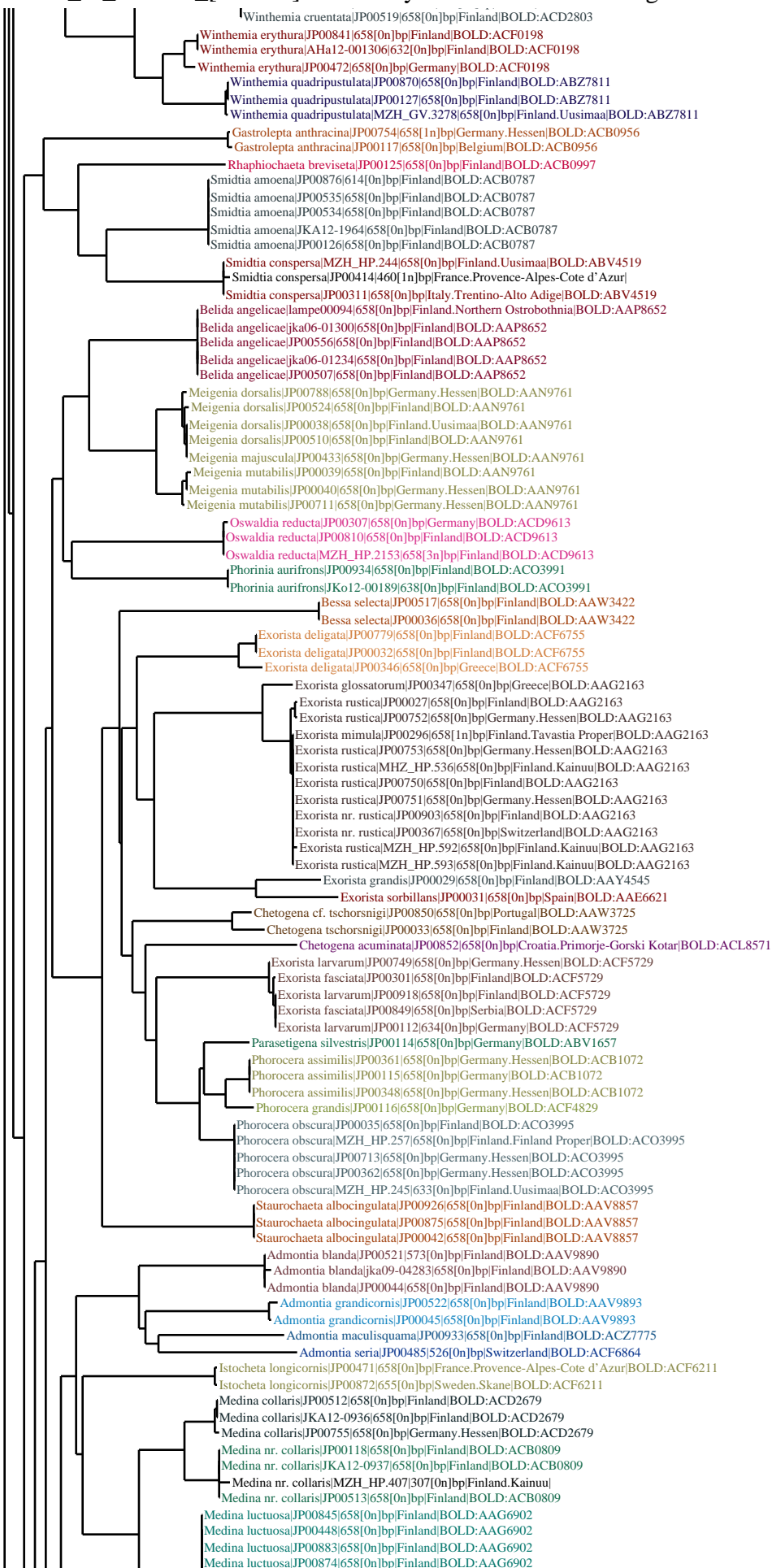

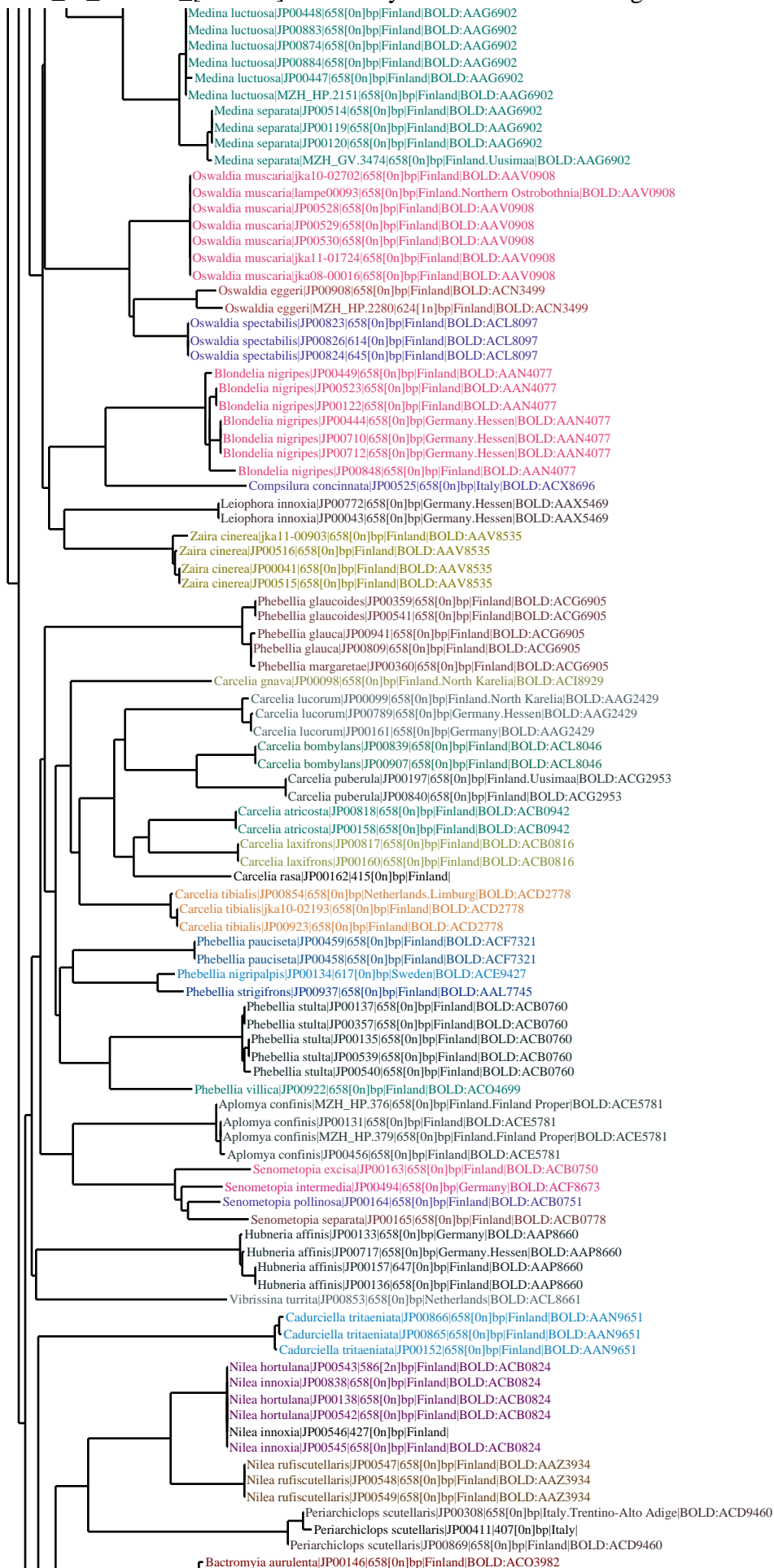

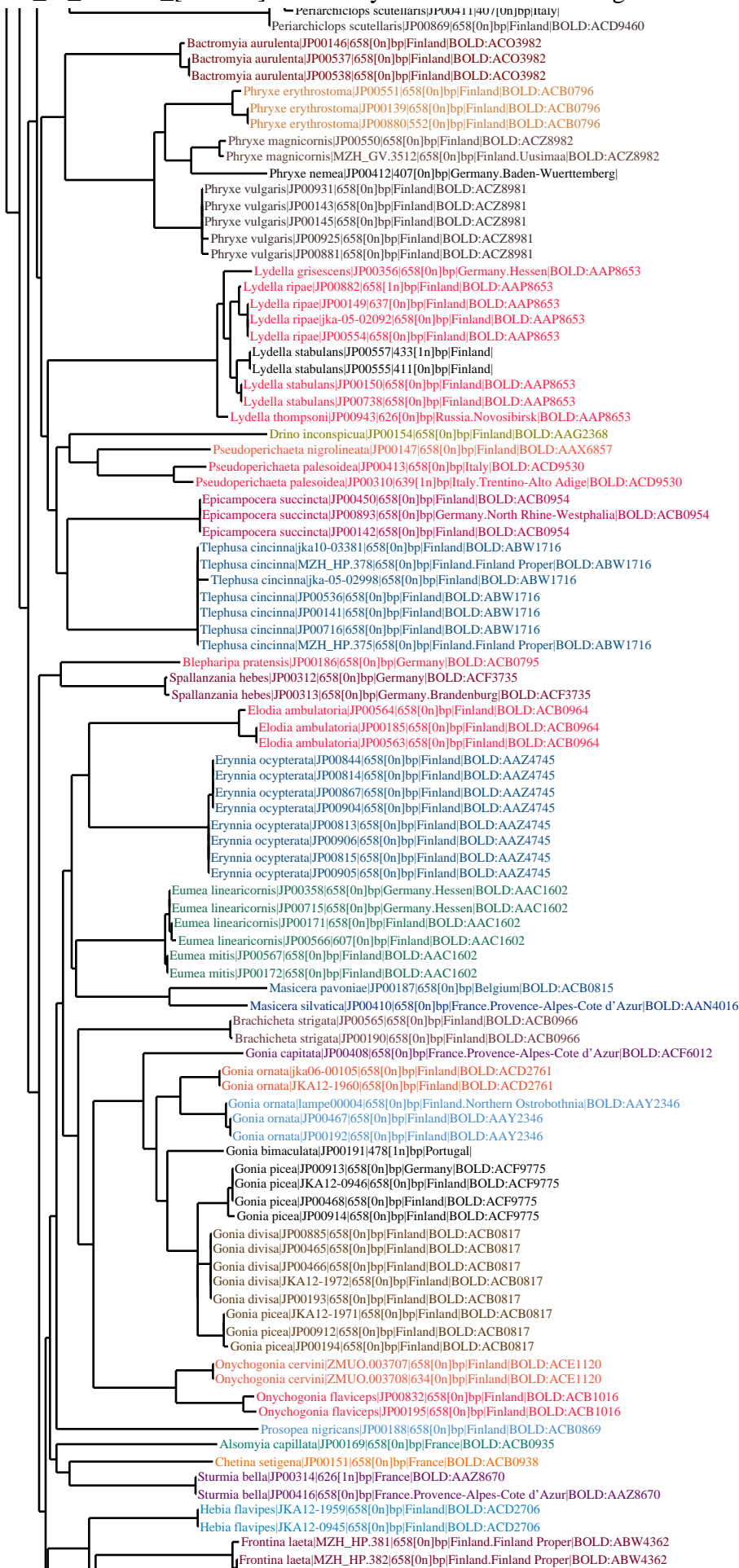

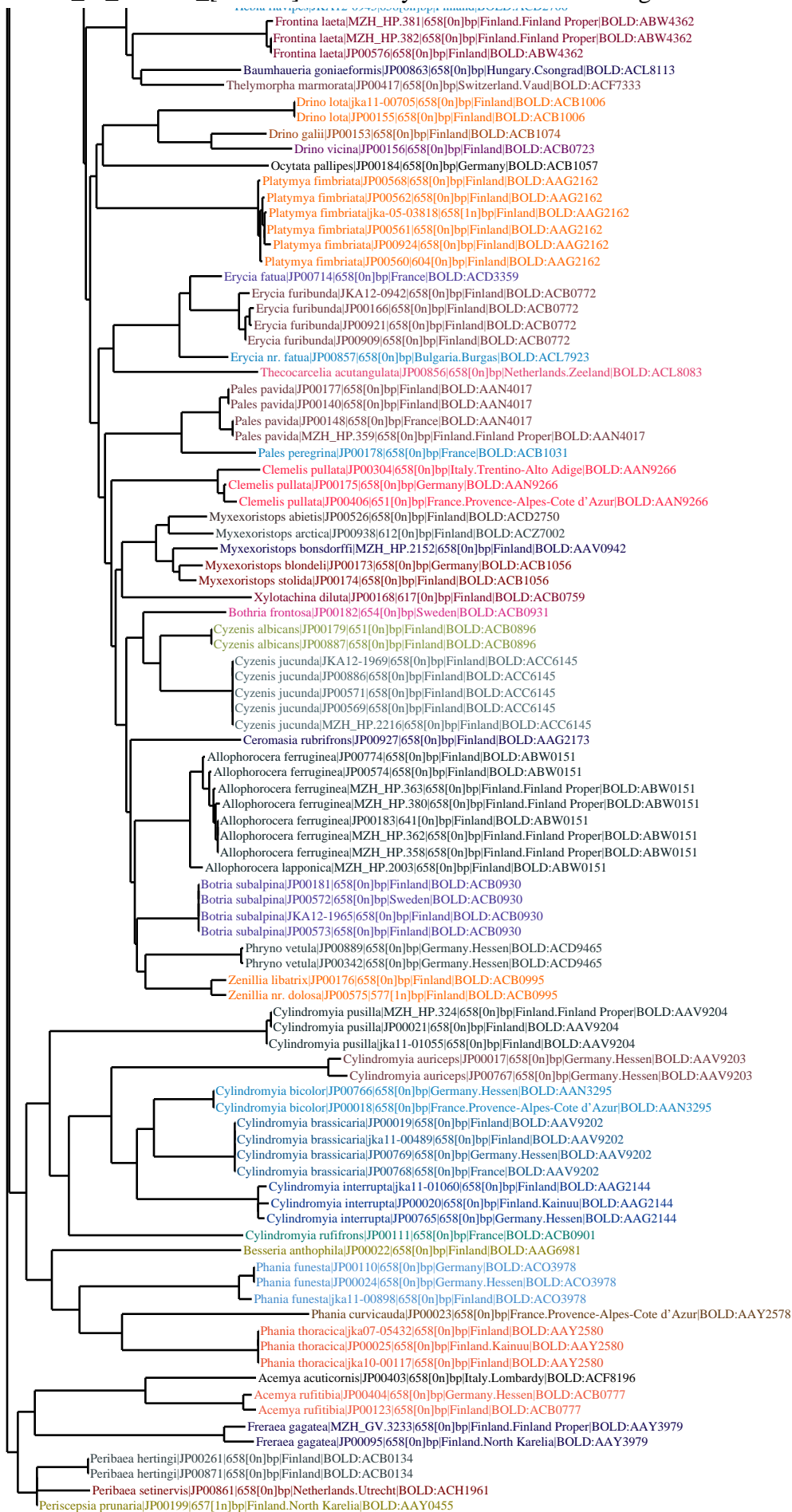

Supplement: S1 Fig — BIN clusters given in different colours. (PDF) [file pone.0164933.s001.pdf]
